# Tryptone-stabilized gold nanoparticles induce unipolar clustering of supernumerary centrosomes and G1 arrest in triple-negative breast cancer cells

**DOI:** 10.1038/s41598-019-55555-3

**Published:** 2019-12-13

**Authors:** J. Grace Nirmala, Manu Lopus

**Affiliations:** School of Biological Sciences, UM-DAE Centre for Excellence in Basic Sciences, University of Mumbai, Vidyanagari Mumbai, 400098 India

**Keywords:** Mechanisms of disease, Mechanism of action

## Abstract

Gold nanoparticles of different sizes, shapes, and decorations exert a variety of effects on biological systems. We report a novel mechanism of action of chemically modified, tryptone-stabilized gold nanoparticles (T-GNPs) in the triple-negative breast cancer (TNBC) cell line, MDA-MB-231. The T-GNPs, synthesized using HAuCl_4_.3H_2_O and tryptone and characterized by an assortment of spectroscopy techniques combined with high-resolution electron microscopy, demonstrated strong antiproliferative and anti-clonogenic potential against MDA-MB-231 cells, arresting them at the G_1_ phase of the cell cycle and promoting apoptosis. The molecular mechanism of action of these particles involved induction of unipolar clustering and hyper amplification of the *supernumerary centrosomes* (a distinctive feature of many tumour cells, including TNBC cells). The clustering was facilitated by microtubules with suppressed dynamicity. Mass spectrometry-assisted proteomic analysis revealed that the T-GNP-induced G_1_ arrest was facilitated, at least in part, by downregulation of ribosome biogenesis pathways. Due to the presence of supernumerary centrosomes in many types of tumour cells, we propose chemical induction of their unipolar clustering as a potential therapeutic strategy.

## Introduction

Gold nanoparticles of different sizes, shapes, and decorations have been investigated for their therapeutic, imaging, and diagnostic potential^1,2^. However, studies exploring the fine details of the molecular-level interactions of gold nanoparticles inside cancer cells, the manifestations of these interactions, and their clinical implications, are poorly understood.

Centrosomes are microtubule-organizing centres in mammalian cells. A normal mammalian cell with division potential typically possesses one centrosome during its non-dividing phase (the ‘interphase’) and duplicates the centrosome during the division phase (the ‘mitotic phase’). However, many cancer cells harbour multiple copies of centrosomes during interphase. For example, ‘supernumerary centrosomes’ are a characteristic feature of the majority of aggressively invasive breast cancer cells, including MDA-MB-231^3,4^. Although the precise role of these supernumerary centrosomes is not clear, they are thought to facilitate tumour metastasis^4^. As two centrosomes (one on each pole) are necessary and sufficient for normal cell division, the numerical aberration of centrosomes poses a serious challenge during cell division. However, the cells with multiple copies of centrosomes cluster them at both the poles to mimic bipolarity -an essential prerequisite for progression of the cell through its division cycle^5^. From a therapeutic perspective, disruption of this bipolarity by ‘declustering’ the centrosomes has been investigated as a potential treatment strategy^6–9^. However, drugs that are known to induce centrosome declustering, such as griseofulvin^10^, are associated with serious side effects including liver damage^11^, necessitating identification of novel drug molecules with reduced side effects or finding alternate centrosome-targeted strategies.

Microtubules are dynamic cytoskeletal polymers in eukaryotic cells that take part in several functions, including the positioning and maintenance of centrosomes and providing structural stability to the cell^12,13^. These cytoskeletal filaments are generated by the reversible addition of a double-subunit protein, tubulin. Once assembled, microtubules exhibit selective stability as per the need of the cell. Interphase microtubules tend to be more stable than mitotic microtubules. The stability of the microtubules is governed by several post-translation modifications. Acetylation of the tubulin subunits of the microtubules, for example, is a sign of persistently stable microtubules^12^. Due to their involvement in vital cellular functions, tubulin and microtubules are targets for several clinically-approved anticancer drugs, including taxanes, ixabepilone, and eribulin mesylate^14^. By as yet poorly understood mechanism(s), breast neoplasms have been found to respond favourably to anti-tubulin agents; all the drugs mentioned above are prescribed chiefly for breast tumours. However, in order to overcome dose-limiting and off-target toxicities that are associated with current chemotherapeutics, it is imperative to develop potential therapeutic candidates that can specifically eliminate cancer cells by exploiting their differential constitution.

Tryptone, a mixture of peptides formed by the digestion of casein by trypsin, stabilizes the gold nanoparticles in solution. The surface modifications of the nanoparticles by tryptone make them stable and reduce the tendancy of the particles to aggregate^15^. As the peptides form the coating over the gold nanoparticles, they render them biocompatible as well^16^. This study reports a novel mechanism of action of the T-GNPs that involves the supernumerary centrosomes of the triple-negative breast cancer (TNBC) cell line, MDA-MB-231.

## Results

### Characterization of the T-GNPs

**Figure 1:**
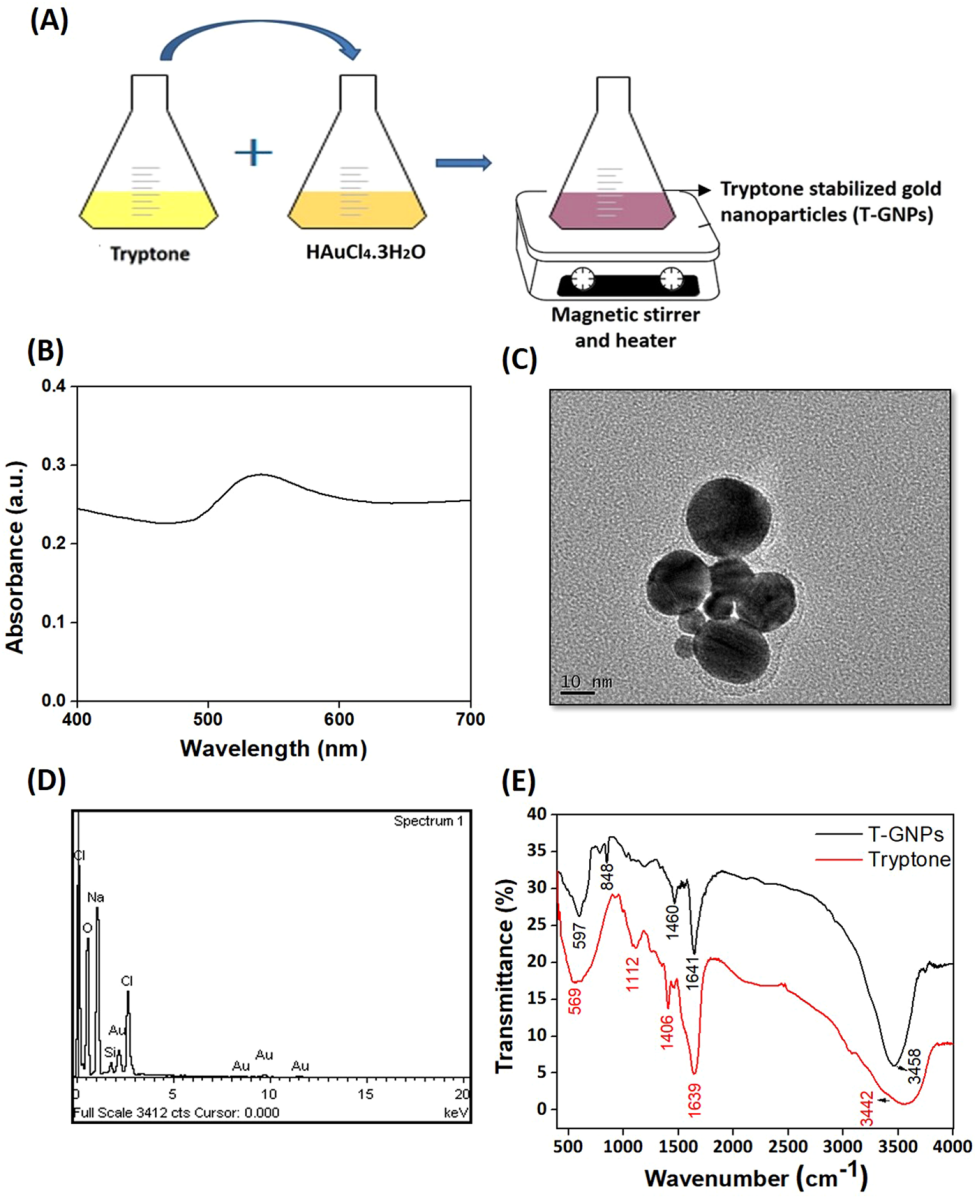
Synthesis and characterization of the T-GNPs. (**A**) Synthesis of the T-GNPs using HAuCl_4_.3H_2_O and tryptone. (**B**) UV-vis absorbance spectra of T-GNPs indicating the presence of the particles. (**C**) Electron micrograph of T-GNPs (**D**) EDX spectrum showing the presence of gold. (**E**) FTIR spectra of tryptone and T-GNPs showing the functional groups.

The T-GNPs used in this study were synthesized from HAuCl_4_.3H_2_O and tryptone (Fig. 1A). The colour change of the solution from light yellow to reddish pink provided the first indication of the formation of nanoparticles. The formation of the T-GNPs and their possible spherical shape were indicated by the absorbance peak at 540 nm (Fig. 1B)^17^. Transmission electron microscopy (TEM) images of the nanoparticles confirmed their spherical shape and their average size distribution (~25 nm) (Fig. 1C). An energy dispersive X-ray (EDX) spectroscopy analysis of the nanoparticles revealed the presence of elemental gold (Au) (Fig. 1D). The functional groups of tryptone involved in the reduction of Au^3+^ and the capping molecules on the synthesized T-GNPs were verified by Fourier-transform infrared (FTIR) analysis (Fig. 1E). The intense broad peaks at 3442 cm^−1^ and 3458 cm^−1^ for tryptone and T-GNPs, respectively, were due to amine N-H stretching vibration. For tryptone, the presence of a strong peak at 1639 cm^−1^ represents –C=C– stretching vibration, and the bands at 1406 cm^−1^ represent CH_2_ bending vibration. The absorption peak at 1112 cm^−1^ could be attributed to C–N stretching vibrations of amine groups. Thus, it was confirmed that tryptone molecules acted as the reducing and capping agents of T-GNPs. The FTIR spectrum after the bioreduction of the T-GNPs showed a sharp peak at 1641 cm^−1^, which represents string C=O stretching vibration of amide groups. The peak at 1460 cm^−1^ is characteristic of medium CH_2_ deformation bending vibration, and the sharp peak at 848 cm^−1^ represents symmetrical C–N–C stretching vibration of amine groups^18^. A size-distribution histogram of the particles as determined by dynamic light scattering (DLS) exhibited hydrodynamic sizes with a mean value for the intensity distribution (Z average), 269 ± 10.0 nm (Supplementary Fig. 1A). The polydispersity index (PdI) of 0.474 substantiated the stability of the T-GNPs (Supplementary Fig. 1B)^19^. Further, the T-GNPs exhibited a negative zeta potential of −18.5 ± 5 mV (Supplementary Fig. 1B), suggesting that the particles had fewer tendencies to aggregate.

### T-GNPs inhibited cell viability, clonogenicity and cell cycle progression, and induced cell death

**Figure 2:**
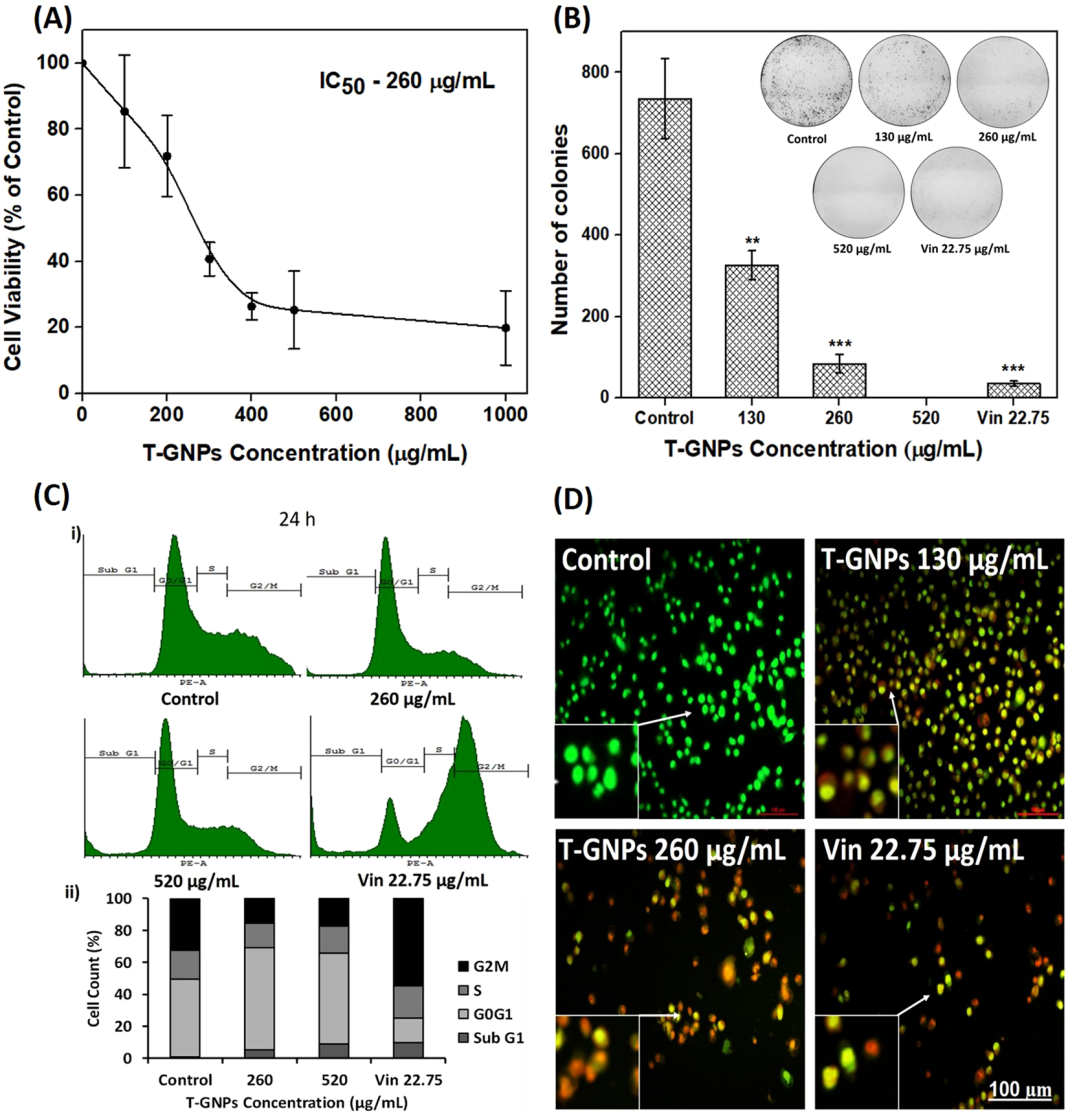
Effects of T-GNPs on MDA-MB-231 cell population. (**A**) Inhibition of cell viability. The cells were exposed to the T-GNPs (100 µg/mL – 1000 µg/mL) and the cell viability was determined using an MTT assay. (**B**). Inhibition of clonogenicity. The T-GNPs inhibited the colony forming ability of the cells grown for eight days after a brief exposure (24 h) to the nanoparticles. (**C**) Cell cycle arrest. The cells were treated with the T-GNPs (260 µg/mL and 520 µg/mL) for 24 h and cell cycle distribution was analyzed using flow cytometry. The results are represented as histograms (**C**i). The graphs (**C**ii) represent the percentage of cells at different phases of the cell cycle. (**D**) Induction of apoptosis. The T-GNPs, at their IC_50_ for cell viability, considerably increased the population of apoptotic (greenish yellow – early apoptosis, and orange – late apoptosis) and dead cells (dark orange to red); (n = 3). For A and B, the results are expressed as mean ± SD (n = 3); **(p < 0.01), and ***(p < 0.001). *Vin*, vinblastine, 22.75 µg/mL (25 µM).

The T-GNPs-treated cells showed concentration-dependent inhibition of cell viability (Fig. 2A). For example, the T-GNPs at 100 µg/mL, 300 µg/mL, and 500 µg/mL inhibited the cell viability by 15%, 59%, and 75%, respectively, yielding a half-maximal inhibitory concentration (IC_50_) of 260 ± 5 µg/mL. The nanoparticles also inhibited the clonogenic propagation of the cells in a concentration-dependent manner (Fig. 2B). For example, 130 µg/mL and 260 µg/mL of the particles inhibited the clonogenicity by 56% and 89%, respectively, whereas 520 µg/mL of the particle nearly completely inhibited the clonogenicity. Vinblastine, used as a control, reduced the number of colonies by 95%. As demonstrated by flow cytometry, the cells treated with T-GNPs for 24 h arrested the cells at G_0_/G_1_ phase (Fig. 2C). Specifically, cells treated with the IC_50_ (260 µg/mL) of T-GNPs for cell viability showed a 31% increase in the G_0_/G_1_ population (from 49% to 64%), compared to the control cells. When the cells were exposed to twice this concentration (520 µg/mL) for 24 h, the sub-G_1_ phase increased substantially, suggesting the accumulation of degraded DNA associated with cell death (Fig. 2C). A 48-h treatment with the T-GNPs showed the accumulation of cells in the sub-G_1_ phase with little or no cells present in other phases (Supplementary Fig. 2), indicating time-dependent increase in cell death. Thus, the T-GNPs are capable of inducing robust G_1_ arrest and promoting their death. Vinblastine, as expected, showed strong G_2_/M arrest (55% cell in G_2_/M, compared to the control). Cell death was confirmed using the DNA-intercalating fluorescent dyes acridine orange and ethidium bromide (AO/EtBr staining). AO can enter the cells through the intact cell membrane and stain the DNA, whereas EtBr can only stain the DNA of cells with compromised membrane integrity^20^. The T-GNPs induced concentration-dependent induction of cell death. The following observations were made: (1) untreated cells were mostly stained green, indicating the predominance of healthy cells, (2) cells in the early stages of apoptosis showed greenish yellow staining, (3) cells in late apoptosis showed orange staining, and (4) dead cells showed dark orange to red fluorescence (Fig. 2D).

### Induction of unipolar clustering of the supernumerary centrosomes by the T-GNPs

**Figure 3:**
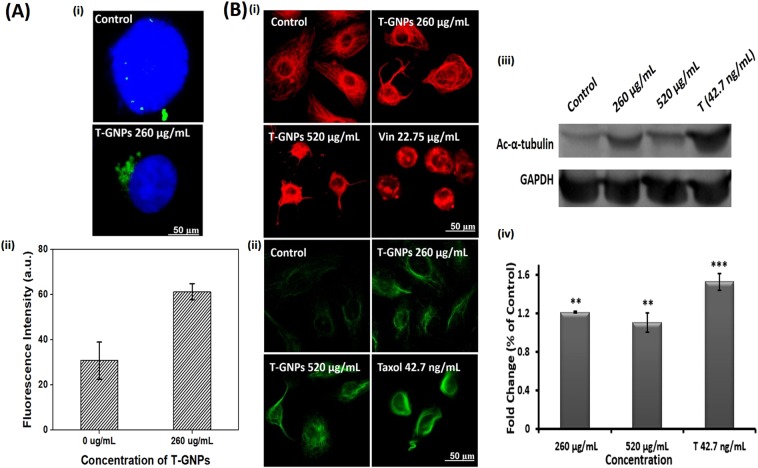
Intracellular effects of the T-GNPs. (**A**.i.) The T-GNPs cluster gamma-tubulin to one pole of the cell. MDA-MB-231 cells treated with IC_50_ (260 µg/mL) of the T-GNPs for cell viability were stained with anti-γ-tubulin antibodies (for centrosomes; green) and Hoechst 33342 (for DNA; blue). (**A**ii) Amplification of the clustered centrosomes. The fluorescence intensities corresponding to centrosome staining were quantitated using Image J software. (**B**i) Bundling and (**B**ii) hyper stabilization of cellular microtubules induced by the T-GNPs. The cells treated with 260 µg/mL or 520 µg/mL of the T-GNPs were visualized using anti-α-tubulin antibodies (red) or anti-acetyl-α-tubulin (green) antibodies. (**B**iii and **B**iv) Increase in the expression levels of acetyl-α-tubulin in the T-GNPs-treated cells. Western blot showing levels of acetylated tubulin in the presence of the T-GNPs (260 µg/mL and 520 µg/mL). The blots were cropped from different parts of the same blot. The full-length blot at different exposure levels is given in supplementary information. For **A**.ii. and **B**iv, the data were statistically significant at ‘**’(*P* < 0.01) and ‘***’(*P* < 0.001), when compared to the controls. The results are expressed as mean ± SD; (n = 3). *Vin*, vinblastine 22.75 µg/mL (25 µM); *T*, taxol, 42.7 ng/mL (50 nM).

Immunostaining of the cells with anti-gamma tubulin antibodies revealed the presence of supernumerary centrosomes in interphase cells (Fig. 3A), which presented as scattered dots, as reported^21^. An average of 5–6 centrosomes per cell were found in untreated cells (Fig. 3Ai). The cells treated with the T-GNPs (260 µg/mL) showed clustering of the centrosomes to one pole (Fig. 3Ai). The centrosomes thus clustered also showed further amplification (Fig. 3Aii).

### Stabilization and bundling of cellular microtubules by the T-GNPs

The cells treated with T-GNPs (260 µg/mL or 520 µg/mL) for 24 h showed extensive bundling of the microtubules (Fig. 3Bi). Vinblastine, that induced mitotic arrest, depolymerized the cellular microtubules. Persistent stabilization of the microtubules was further revealed by immunostaining with anti-α-acetyl tubulin antibodies (Fig. 3Bii) and immunoblotting of the treated cells’ proteins (Fig. 3Biii,iv). In both cases, the control used, Taxol, also showed elevated levels of acetylated tubulin. Taxol revealed extensive acetylation of the microtubules. Total α-tubulin was also estimated by immunostaining with anti- α-tubulin antibodies (Supplementary Fig. 3).

**Figure 4:**
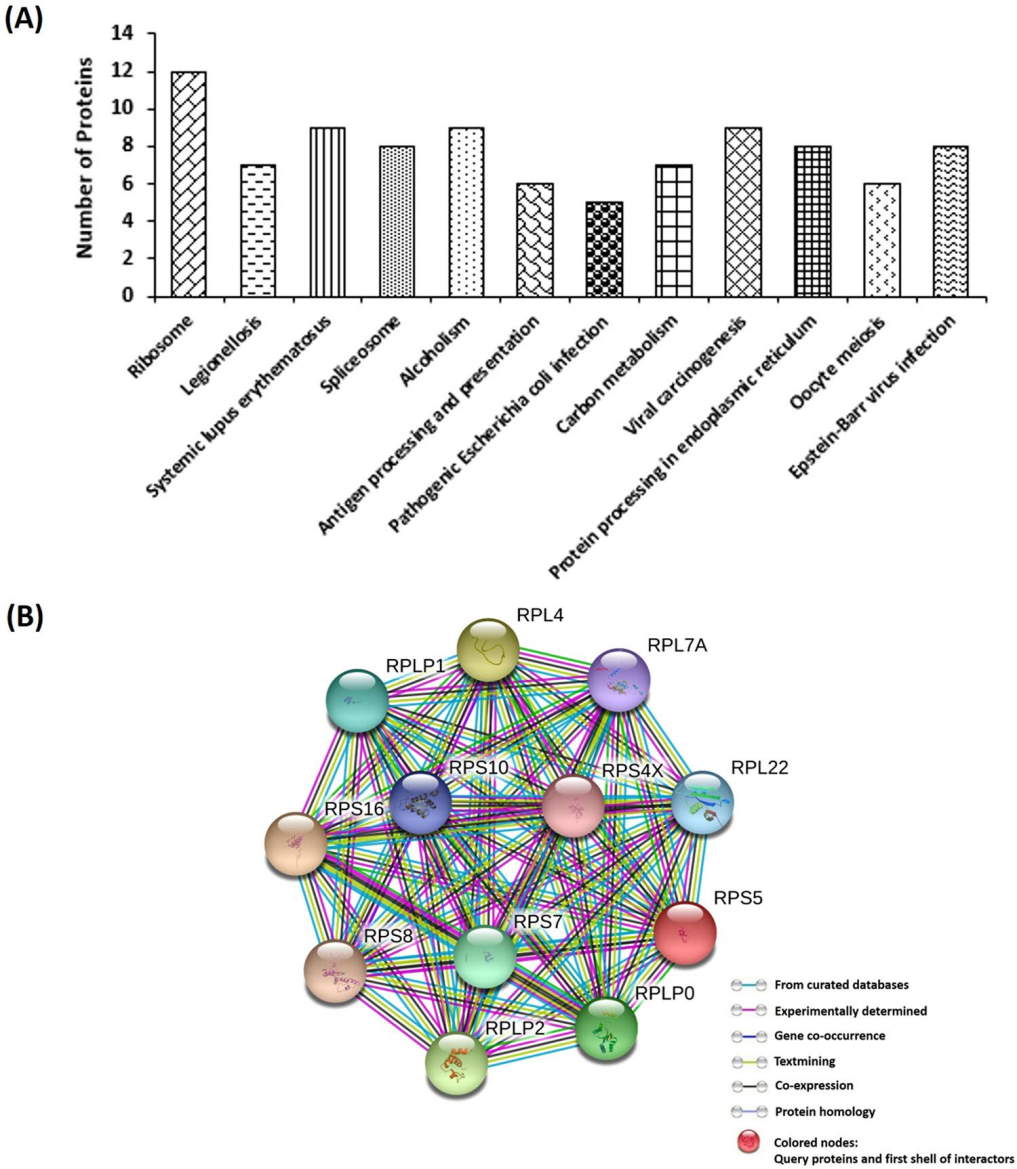
Proteomic analysis of differentially expressed proteins in T-GNPs treated MDA-MB-231 cells. (**A**) Bar graphs showing KEGG pathways of differentially-expressed proteins. (**B**) The protein interaction network of the differentially-expressed proteins of the ribosome pathway, as analyzed by STRING 10.5 database. Each node represents the proteins and the coloured lines represent the protein-protein interactions.

In order to further understand the T-GNPs’ mechanism of action on the TNBC cells, proteomic analyses were performed. Protein identification in untreated and treated cells was performed as per established criteria^22^. Fasta files from the Human Proteome database (downloaded from the UniProt) were used for protein searches, and a list of identified proteins was generated. The changes in protein expression levels in the treated cells compared to the control cells were quantified using label-free quantification methods and are given in Supplementary Table 1. R^2^ values for two repeats were more than 0.99 in the untreated and treated groups (one-way analysis of variance). The interaction network of the 155 proteins that were downregulated by the T-GNPs were analyzed using the Search Tool for the Retrieval of Interacting Genes/Proteins (STRING) database (Supplementary Fig. 4A). The proteins that were thus differentially expressed were subjected to gene ontology analysis (GO), which identified 134 biological processes (BP), 81 cellular components (CC), and 68 molecular functions (MF) that were enriched for the dataset. Among these, 56 MFs, 61 CCs, and 98 BPs showed *p* < 0.05 (Supplementary Fig. 4B–D). Kyoto Encyclopaedia of Genes and Genomes (KEGG) pathway analysis showed that the ribosome biogenesis pathway was the most distinctly downregulated, with 12 proteins (Fig. 4A,B). The spliceosome pathway showed downregulation with eight proteins, and carbon metabolism with seven proteins (Fig. 4A).

### T-GNPs elevated ROS production and induced loss of mitochondrial membrane potential

T-GNPs significantly (*p* < 0.01) elevated the levels of ROS inside the cells. Specifically, compared to the untreated cells, the cells treated with 130 µg/mL and 260 µg/mL of the T-GNPs increased the ROS level by 2.6-fold and 3-fold, respectively, as measured by their relative fluorescence intensities (Supplementary Fig. 5A). To confirm whether the increased ROS production observed in the treated cells was mediated by the T-GNPs, we pre-treated the cells with N-acetyl cysteine (NAC) and then with the T-GNPs. As shown (Supplementary Fig. 5A), the increase in ROS production in the T-GNPs-treated cells was significantly quenched when the cells were pre-treated with NAC. H_2_O_2,_ a known inducer of ROS, was used as the positive control which increased the ROS by 5-fold (Supplementary Fig. 5A). As the increased levels of ROS can adversely affect the integrity of the mitochondrial membrane potential, we next examined the effect of the particles on the potential (*Δψ*m) using the membrane permeable, a lipophilic dye, Rhodamine 123. Rhodamine 123 exhibited a heterogeneous staining pattern in the untreated cells with both red and green fluorescence coexisting in the same cell (Supplementary Fig. 5B). Cells treated with 130 µg/mL of T-GNPs showed a considerable loss of the membrane potential as evidenced by the marked reduction in red fluorescence and the increase in green fluorescence. When the concentration was doubled, a near-complete absence of the red fluorescence was observed, indicating substantial loss of the mitochondrial membrane potential.

## Discussion and Conclusion

The unique effects of gold nanoparticles, when exposed to the cellular milieu, are just beginning to be fully comprehended. This study used T-GNPs (~25 nm) to unravel a novel mechanism of action these particles against one of the most aggressively metastatic breast cancer cell lines, MDA-MB-231. The mechanism involves unipolar clustering and hyper amplification of supernumerary centrosomes and robust G_1_ arrest, leading to cell death.

We began our investigation by assessing the antiproliferative potential of the T-GNPs against the cells. After verifying that the T-GNPs at microgram quantities inhibit cell viability by retarding the cells’ proliferative, clonogenic, and cycling potential (Fig. 2), we set out to investigate the particles’ molecular mechanism of action. One characteristic feature of TNBC cells is the presence of supernumerary centrosomes^21^. This numerical abundance of centrosomes has been thought to assist the cells in reorganizing their cytoskeleton, facilitating survival and metastasis^4^. Therefore, we first examined if the nanoparticles have any effect on these supernumerary centrosomes. Using anti-gamma tubulin antibodies, we identified a novel reorganization of these centrosomes in the treated cells. Unlike the untreated TNBC cells that displayed scattered centrosomes (Fig. 3A), the treated cells exhibited unipolar clustering and amplification of the centrosomes (Fig. 3A). Since the positioning of centrosomes is governed chiefly by the forces transmitted by microtubules^13^, we next examined whether the particles altered the structure or stability of the microtubule network. As revealed by fluorescence microscopy, the T-GNPs induced bundling of the microtubules (Fig. 3Bi). Further, the particles-treated microtubules displayed enhanced acetylation indicating suppression of their dynamicity (Fig. 3Bii)^23^. Specifically, alpha-tubulin can get acetylated at its lysine 40. Whether acetylation is the cause or consequence of microtubule stabilization is still unclear. Nevertheless, enhanced acetylation pattern indicate the presence of persistently stable microtubules^23^. Next, we examined whether the particles interacted directly with tubulin (the building block protein of microtubules) to rule out the possibility that the observed bundling of the microtubules was a secondary, indirect effect of the T-GNPs on microtubules. The direct binding of the particles to tubulin was verified using a tryptophan fluorescence-quenching assay (Supplementary Fig. 6). Next to be answered was how the stabilization and bundling of the microtubules induced unipolar clustering of the scattered centrosomes. In an elegant study on ciliary functions^24^, Pitavel and colleagues demonstrated that hyperstable microtubules exert pushing forces that can propel centrosomes towards one pole. Therefore, it can assumed that the particles propelled and clustered the centrosomes to one pole by interfering with the structure and dynamics of the microtubules and thereby, inducing their hyper stabilization (Fig. 3B). The centrosomes thus clustered were apparently amplified as well (Fig. 3Aii), which could be due to the oxidative stress exerted by the particles (Supplementary Fig. 5A)^25^ complimented via loss of mitochondrial membrane potential (Supplementary Fig. 5B)^26^. Finally, how the cells lost their cycling potential at the G_1_ stage was investigated. Although the loss of mitochondrial function (Supplementary Fig. 5B) can promote G_1_ arrest^27^, we sought to uncover additional mechanisms that might have facilitated the arrest with the help of mass spectrometry-assisted proteomics analysis. The analyses suggested that the downregulation of the ribosomal biogenesis pathway (Fig. 4A) played a key role in inducing G_1_ arrest^28,29^. Drugs that target ribosome biogenesis pathway have been found to be effective in retarding cancer cell proliferation by inhibiting pre-rRNA processing, rRNA synthesis and interfering with translation. Several chemotherapeutic agents target ribosomes biogenesis as an effective antiproliferative strategy^30,31^. Here, as revealed by KEGG pathway analysis, at least twelve ribosomal proteins with close interactions to each other were downregulated in cells that were exposed to the T-GNPs (Fig. 4A,B). The proteins were the ribosomal P-complex proteins (RPLP0 [for *ribosomal protein lateral stalk subunit* P0], RPLP1, RPLP2), RPL4, RPL7A, RPL22, RPS4X, RPS5, RPS7, RPS8, RPS10, and RPS16. Notably, downregulation of P-complex proteins, such as RPLP0, is known to promote G_1_ arrest^32^. In fact, depletion of ribosomal proteins from either ribosomal subunit can elicit G1 arrest^33^. Other ribosomal proteins that were downregulated by the T-GNPs also hold therapeutic significance for cancer. For example, depletion of the RPS4X protein in SK-OV-3 cells can strongly retard their proliferative potential^34^. In addition, there is a relation between elevated levels of ROS (Supplementary Fig. 5A) and suppression of ribosomal function. Specifically, oxidative stress is known to retard global protein synthesis, caused largely by a slower rate of ribosomal runoff due to its inhibitory effect on translation elongation or termination^35^. Further, an elegant study by Willi and colleagues showed that oxidative stress damages rRNA inside the ribosome^36^.

In summary, this study elucidated a novel mechanism of action of gold nanoparticles in cancer cells, which is characterized by unipolar clustering of supernumerary centrosomes. The clustering was facilitated by loss of microtubule dynamics. The clustering, coupled with robust G_1_ arrest promoted by the downregulation of ribosomal biogenesis pathway, contributed to the cell death.

## Materials and Methods

### Materials

Gold (III) chloride trihydrate (HAuCl_4_.3H_2_O), dichlorofluorescein diacetate (DCFH-DA), Rhodamine 123, protease inhibitor cocktail, phenylmethanesulfonyl fluoride (PMSF), propidium iodide (PI), formaldehyde, Taxol, ethidium bromide, guanosine-5′-triphosphate (GTP), glutamate, piperazine- N, N′-bis (2-ethane sulfonic acid) (Pipes), magnesium sulfate (MgSO_4_), ethylene glycol tetraacetic acid (EGTA), vinblastine, dithiothreitol (DTT), iodoacetamide (IAA), ammonium bicarbonate (ABC), trifluoroacetic acid (TFA), formic acid, sodium chloride, Triton X-100, and sodium deoxycholate were procured from Sigma (St. Louis, MO). Dulbecco’s Modified Eagle’s Medium (DMEM), fetal bovine serum (FBS), trypsin-EDTA (0.25%) solution, penicillin, streptomycin, bovine serum albumin (BSA), horse serum, (3-(4,5- dimethylthiazol-2-yl)-2,5-diphenyltetrazolium bromide) (MTT), ribonuclease A, dimethyl sulfoxide (DMSO), acetonitrile, and HPLC-grade water were purchased from Himedia, India. Tryptone, Tris buffer, acridine orange, and crystal violet were from Sisco Research Laboratories (SRL, Bangalore, India). Phosphatase inhibitor, trypsin-protease, and Prolong Gold anti-fade reagent were obtained from Thermo Scientific (Waltham, Massachusetts). Urea was obtained from Rankem, India. All other chemicals and solvents were also of analytical grade and highest purity.

### Synthesis and characterization of the T-GNPs

Tryptone-stabilized gold nanoparticles (T-GNPs) were synthesized as follows. Gold (III) chloride trihydrate (1 mM) was mixed with tryptone (1 mg/mL; pH 12) by continuous stirring at 25 °C. The solution was then heated on a hot plate for 15 min at 100 °C with continuous stirring. The sample was then centrifuged at 14,000 rpm (26, 200 × g) for 30 min in a Sorvall™ Stratos Biofuge centrifuge (Thermo Scientific, Waltham, MA). The sedimented gold nanoparticle were then washed thrice with deionized water, and freeze-dried using FreeZ-Zone freeze-dry system (Labconco^(R)^, Kansas City, MO) to obtain powdered gold nanoparticles.

The presence of the synthesized T-GNPs was verified first by UV-visible spectrophotometry (Infinite^®^ 200 PRO, Tecan, Switzerland)^17^ by taking the absorbance of the samples (400 nm–700 nm). The size and the shape of the particles were examined using a JEOL JEM-2100F transmission electron microscope (JEOL, Tokyo, Japan)^37^. To examine the presence of elemental gold, energy dispersive X-ray spectroscopy (EDX) (INCA, Oxford Instruments, UK) was performed. To identify the functional groups on the surface of the nanoparticles, Fourier-transform infrared (FTIR) spectral analyses were performed in an FTIR spectrophotometer (Spectrum RX1, Perkin Elmer, USA) in transmission mode (400 cm–4000 cm^−1^) at a resolution of 4 cm^−1 ^^37^. The surface charge, stability, size distribution and the average hydrodynamic diameter of the T-GNPs were obtained using a Zetasizer Nano-ZS90 size analyzer (Malvern Instruments Ltd, Worcestershire, UK)^37^.

### Cell culture

MDA-MB-231 (triple-negative breast cancer) cells were obtained from American Type Culture Collection (ATCC, Manassas, VA). The cells, at their 21^st^ passage, were cultured in DMEM supplemented with 10% heat-inactivated FBS, and penicillin (100 U/mL)/streptomycin (0.1 mg/mL) solution. The cells were maintained in a humidified atmosphere at 37 °C and 5% CO_2_ in a Forma SteriCycle incubator (Thermo Scientific, Waltham, MA). The cells were of low passage number. By using a MycoAlert mycoplasma detection kit (Lonza, Basel, Switzerland), they were determined to be free of mycoplasma.

### Cell viability and colony-formation assays

The effect of the T-GNPs on the cell viability was assessed using an MTT assay^38^. In brief, the cells were seeded at a density of 5000 cells/well in a 96-well plate and incubated for 24 h. Next day, the cells were treated with different concentrations of the T-GNPs (100 µg/mL–1000 µg/mL) for 24 h at 37 °C. After the specified time point, MTT (5 mg/mL) in phosphate-buffered saline (PBS) was added to each well and incubated for 4 h at 37 °C. The formazan crystals formed by the viable cells were dissolved in DMSO. Absorbance measurements were carried in a microplate reader (570 nm; TECAN infinite 200 PRO; Tecan, Switzerland). The experiment was performed three times in triplicates. For the colony-formation assay, the cells were plated in each well of a 6-well plate at 1000 cells/mL and were allowed to adhere for 24 h. They were then treated with 130 µg/mL, 260 µg/mL, and 520 µg/mL of the T-GNPs for 24 h. After the specified time point, the particles-containing media was replaced with fresh, complete media and the cells were grown in it for eight days with one media change on the fourth day. The colonies formed were fixed with 3.7% formaldehyde (37 °C; 15 min) and stained with crystal violet (0.5% (w/v); 1 h, 25 °C). The wells were then washed with distilled water, air-dried, and the colonies were enumerated using Image J software, (National Institutes of Health, USA). The experiment was repeated at least two times.

### Cell cycle analysis

The cells were treated with the T-GNPs (260 µg/mL and 520 µg/mL) for 24 h or 48 h. After the incubation, the adherent and floating cells were collected, washed with 1X PBS and fixed in chilled ethanol (70%) for 12 h at −20 °C. After RNase A (100 µg/mL) treatment of the fixed cells for 10 min, they were incubated with propidium iodide (50 µg/mL; dissolved in 0.1% Triton X-100). The samples analyzed in a BD FACS Aria (BD Bioscience, San Jose, CA) equipped with a FACS DIVA software. The percentage of cells in each phase of the cycle was determined using FlowJo software (BD Bioscience). Further, the number of cells present in each phase of the cycle (G_0_/G_1_, S and G_2_/M) was determined using Cyflogic software (version 1.2.1, Cyflo Ltd., Turku, Finland).

### Detection of apoptosis

Induction of cell death was studied as reported earlier^39^. Briefly, the cells were seeded in 12-well plates and grown for 24 h. The next day, they were treated with the T-GNPs (130 µg/mL or 260 µg/mL) for an additional 24 h. The cells were collected by trypsinization and were washed with 1X PBS. The cell suspension was then incubated with 1 µL of AO/EtBr dye (100 mg/mL each) in PBS. The stained cells were visualized under a Nikon Eclipse 90i fluorescence microscope (Nikon, Tokyo, Japan).

### Visualization of centrosomes, microtubules, and acetylated microtubules

For immunofluorescence visualization of the centrosomes, the cells were stained with anti-gamma tubulin antibodies. Briefly, the cells (5 × 10^4^ cells/mL) were seeded on surface-treated coverslips and grown overnight in 12-well plates. The cells were then treated with the T-GNPs (0 µg/mL or 260 µg/mL) for 24 h and fixed in 3.7% formaldehyde (37 °C, 20 min). The cells were then permeabilized using absolute, chilled methanol at (4 °C, 15 min). Non-specific binding sites were blocked using 5% horse serum (25 °C, 1 h) in a humidified chamber. The cells were stained subsequently with FITC-conjugated γ-tubulin antibodies (Biorbyt, CA, USA; 1:200 dilution; 25 °C, 2 h). After incubating with the antibodies, they were washed with 1X PBS and incubated with Hoechst 33342 (Molecular Probes, Eugene, OR; 1:1000 dilution; 25 °C, 10 min,) to visualize the DNA. To examine the effect of T-GNPs on the cellular microtubule, the cells grown as mentioned above were treated with the T-GNPs (260 µg/mL, 520 µg/mL i.e., IC_50_ and 2 × IC_50_ for the cell viability, respectively) for 24 h, fixed, permeabilized, and the non-specific binding sites were blocked, as mentioned. They were then stained first with anti-α-tubulin antibodies (Sigma, 1:300 dilution; 25 °C, 1 h) and then with Alexa-568-conjugated goat anti-mouse antibodies (Molecular Probes) for the same duration and the temperature. To observe acetylation patterns of the treated microtubules, the cells grown and treated with the nanoparticles (260 µg/mL, 520 µg/mL) were stained with anti-acetyl-α-tubulin (Lys40) antibodies (Cell Signalling Technology, MA; 1: 200 dilution; 25 °C, 2 h) and then with FITC-conjugated donkey anti-rabbit antibodies (Santa Cruz Biotechnology, TX; 1: 1000; 25 °C, 2 h). The coverslips were washed with PBS and mounted on slides using Prolong antifade reagent. The slides were then observed under Nikon 90i fluorescent microscope (40 × magnification, N.A., 0.75) equipped with NIS- Elements BR 3.2 software.

### Western blot

The cells (1 × 10^6^ cells/mL) were seeded in 100-mm cell culture dishes and grown overnight. They were then treated with the T-GNPs (260 µg/mL or 520 µg/mL) for 24 h and lysed in Radioimmunoprecipitation assay buffer [RIPA buffer: sodium chloride (150 mM), Triton X-100 (1%), sodium deoxycholate (0.5%), SDS (0.1%), Tris-HCl (50 mM; pH, 8.0)], supplemented with protease inhibitor cocktail (1%) and PMSF (1 mM). After the cell lysis, the samples were centrifuged and the supernatants were carefully collected and stored at −80 °C until use. The levels of acetylated tubulin and total alpha tubulin were visualized and estimated by immunoblotting using rabbit monoclonal anti-acetyl-α-tubulin (Lys40) antibodies (1:1000 dilutions) and mouse anti-α-tubulin antibodies (1:1000 dilutions) respectively. Glyceraldehyde-3-phosphate dehydrogenase (GAPDH; 1:1000 dilutions; Cell Signaling Technology, Danvers, MA) was used as the loading control. The blots were developed using SuperSignal™ West Pico Chemiluminescent Substrate (Thermo Fisher Scientific) and imaged in a ChemiDoc system (BioRad, Hercules, CA). The band intensities were assessed by ImageJ software.

### Mass spectrometry

For mass-spectrometry-assisted proteomics analysis, the cells, grown in the presence of 260 µg/mL of the particles for 24 h, were centrifuged at 2000 rpm (25 °C, 5 min). Protein extraction was carried out using the lysis buffer (20 mM Tris-HCl, 150 mM NaCl, 1% Triton X-100, 0.5% sodium deoxycholate, 1 mM DTT, 1 X protease inhibitor cocktail, 1 X phosphatase inhibitor cocktail).

#### Precipitation, reduction, alkylation, and digestion of the proteins

The extracted protein samples (control and treated) were precipitated by acetone precipitation^40^. The protein pellets thus obtained were dissolved by adding 8 M urea. The samples were then reduced by the addition of 100 mM DTT and heated in a dry bath at 90 °C for 15 min. After cooling the samples, they were alkylated by adding 200 mM IAA and incubated in the dark (25 °C, 15 min). ABC (100 mM) was then added and proteins were digested with 1 mg/mL trypsin protease (37 °C, 16 h). The reaction was stopped by addition of concentrated TFA. The peptides were then dried using vacuum centrifugation for 24 h, and dissolved in 0.1% TFA for the MS-analysis.

#### Peptide separation and identification

The samples (5 µL, each) were analyzed using a High-Performance Chip (Chip ID**:** G4240–62030) connected to Agilent 1260 infinity HPLC-Chip/MS system (Agilent Technologies, Santa Clara, CA). Charged peptides from the HPLC-Chip system were directly infused into mass-spectrometer for detection, as reported^41^.

#### Spectra analysis, protein database searches and relative quantification

Agilent Mass Hunter software (Mass Hunter Qualitative Analysis B.08.00 Service Pack 1 (SP1)), was used for data acquisition and analysis of total ion chromatograms. Protein searches were carried out using Morpheus software (Howell, MI) with reference to human proteome database^22^. Summed mass spectra from each chromatogram were analyzed manually for accurate identification. *Protein data analysis***:** The differentially-expressed proteins were subjected to pathway enrichment, protein-protein interaction (PPI) analysis and gene ontology (GO) analysis. The analyses were performed using DAVID (for the *d*atabase for *a*nnotation, *v*isualization and *i*ntegrated *d*iscovery) bioinformatics resources 6.8^42,43^ and involved molecular function enrichment (MF), cell component enrichment (CC), biological process enrichment (BP), and Kyoto Encyclopedia of genes and genomes (KEGG) pathway enrichment. Significantly modulated network nodes were reported using the protein dataset^44^. The protein-protein interactions (PPI) were analyzed using the Search Tool for the Retrieval of Interacting Genes/Proteins (STRING/P) database (version 10.5).

### Purification of tubulin

PEM buffer (50 mM Pipes, 3 mM MgSO4, 1 mM EGTA, pH 6.8) was used for the isolation and purification of tubulin from goat brain through temperature and GTP-dependent multiple polymerization and depolymerization cycles, as reported^38^ and kept at −80 °C.

### Tryptophan-quenching assay

Tubulin (2 μM) was incubated in the absence or presence of the T-GNPs (500 µg/mL) in a water-circulating bath (35 °C; 45 mins). After the incubation, the samples excited at 295 nm, and the emission spectra in the range 310 nm–400 nm were obtained. A FlouroMax® 4 spectrofluorometer (Horiba Scientific, Edison, NJ) supported by FluorEssence 3.5 software was used for the spectrofluorimetric titrations^45^.

### Measurement of reactive oxygen species

The cells (5 × 10^4^ cells/mL), grown in 12-well plates and treated with the T-GNPs (130 µg/mL or 260 µg/mL), H_2_O_2_ (400 µM), or N-acetyl cysteine (NAC, 16.3 µg/mL) were examined for evidence of ROS generation after incubating them with DCFH-DA (2.4 mg/mL), as reported earlier^46^. The intensity of the DCF fluorescence was measured using Nikon Eclipse 90i fluorescence microscope and the images were analyzed using Image J software. The fluorescence intensity corresponding to ROS level were quantitated using Image J software. At least 200 cells were counted and their fluorescent intensity was recorded and calculated.

Corrected total cell fluorescence (CTCF).

$${\rm{CTCF}}={\rm{Integrated}}\,{\rm{Density}}-({\rm{Area}}\,{\rm{of}}\,{\rm{selected}}\,{\rm{cell}}\,{\rm{\times }}\,{\rm{Mean}}\,{\rm{fluorescence}}\,{\rm{of}}\,{\rm{background}}).$$

### Analysis of mitochondrial membrane potential

The cells (5 × 10^4^ cells/mL) grown on the poly-L-lysine-coated coverslips in 12-well plates were treated with the T-GNPs (130 µg/mL or 260 µg/mL) and visualized for evidence for loss of mitochondrial membrane potential using Rhodamine 123, as described earlier^47^.

### Statistical analysis

Values are expressed as mean ± standard deviation (SD). Statistical analysis was performed using GraphPad Instat software (San Diego, CA) using one-way analysis of variance (ANOVA). The values were considered statistically significant, if the p-value was <0.05.

## Supplementary information

Supplementary Information

## Data Availability

The datasets generated during and/or analysed during the current study are available from the corresponding author on reasonable request.
