# Supplementary material for: Tryptone-stabilized gold nanoparticles induce unipolar clustering of supernumerary centrosomes and G1 arrest in triple-negative breast cancer cells
Source: Sci Rep. 2019 Dec 13;9:19126. doi: 10.1038/s41598-019-55555-3 (PMC6911093; doi:10.1038/s41598-019-55555-3)
Supplement: Supplementary file 1 — Supplementary Information [file 41598_2019_55555_MOESM1_ESM.docx]

**Supporting Information**

**Tryptone-stabilized gold nanoparticles induce unipolar clustering of supernumerary centrosomes and G1 arrest in triple-negative breast cancer cells**

**J. Grace Nirmala and Manu Lopus***

School of Biological Sciences, UM-DAE Centre for Excellence in Basic Sciences, Vidyanagari, Mumbai -400098, India

**Figure Legends for Supplementary Figures**

**Supplementary Figure 1. (A) Particle size distribution of the T-GNPs showing an average hydrodynamic radius of ~270 nm (B) Zeta potential analysis of the T-GNPs indicating the stability of the particles**

**Supplementary Figure 2 (i&ii). Cell cycle distribution of the cells treated with the T-GNPs for a longer duration (48h) showing widespread cell death.**

**Supplementary Figure 3. Expression levels of total α-tubulin in the T-GNPs-treated cells.**Western blot showing levels of α-tubulin in the presence of the T-GNPs (260 µg/mL and 520 µg/mL). The full-length blots are given in supplementary information. The blots were cropped from different parts of the same blot. The results are expressed as mean ±SD; (n = 3). *T*, taxol – 42.7 ng/mL (50 nM).

**Supplementary Figure 4. (A) The protein-interaction network of the differentially expressed-proteins using STRING 10.5 database.** The interaction network contains 155 identified differentially expressed proteins based on evidence with different types and also indicates the clustered signal pathways in the network analysis. **(B-D) Gene Ontology classifications (GO term: molecular function, cellular components, biological processes) of differentially expressed proteins**

**Supplementary Figure 5. A. The T-GNPs enhanced intracellular ROS.** As visualized (**i**) and quantified (**ii**) using H_2_DCF-DA, the T-GNPs enhanced ROS levels inside the cells (indicated in green). *NAC*- N-acetyl cysteine, *Vin*, Vinblastine. The results are expressed as mean ± SD (n = 3). ‘**’ and ‘***’ represents statistical significance compared to the control group (*p*<0.01) and (*p*<0.001), respectively. **(B) Loss of mitochondrial membrane potential induced by the particles as indicated by Rhodamine 123**. The results were obtained from three independent experiments. Loss of the membrane potential was evidenced from an increase in the green fluorescence and reduction in red fluorescence (also see,^6^). *Vin*, vinblastine – 22.75 µg/mL (25 µM).

**Supplementary Figure 6.** Tryptophan-quenching assay showing a direct interaction of the particles with tubulin. The graph represents one of the three independent experiments (n = 3)

Table S1: MS identification of differentially expressed proteins in the T-GNPs-treated cells

**Supplementary Figures**

**Figure S1**


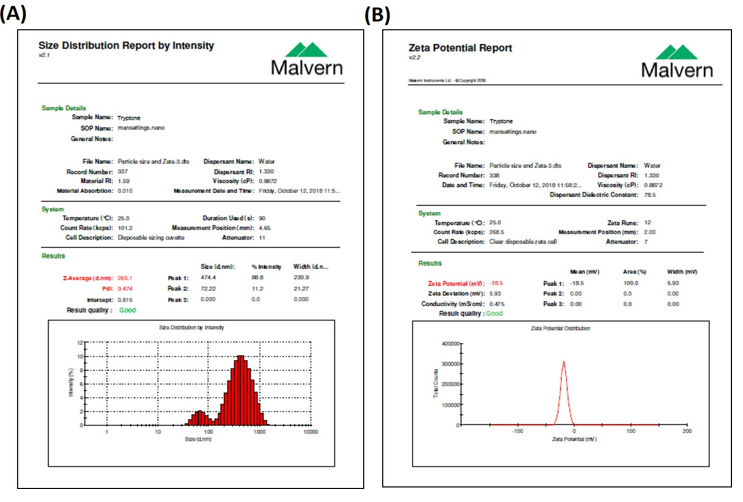


**Figure S2**


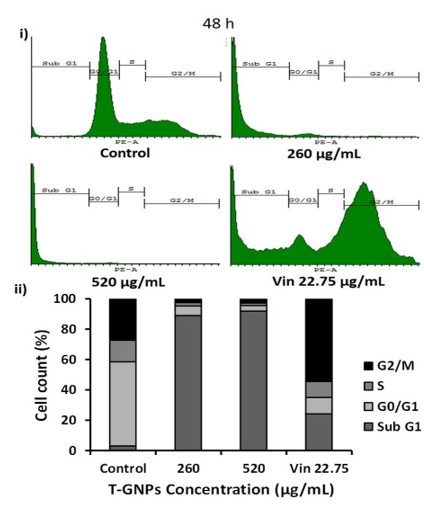


**Figure S3**

**
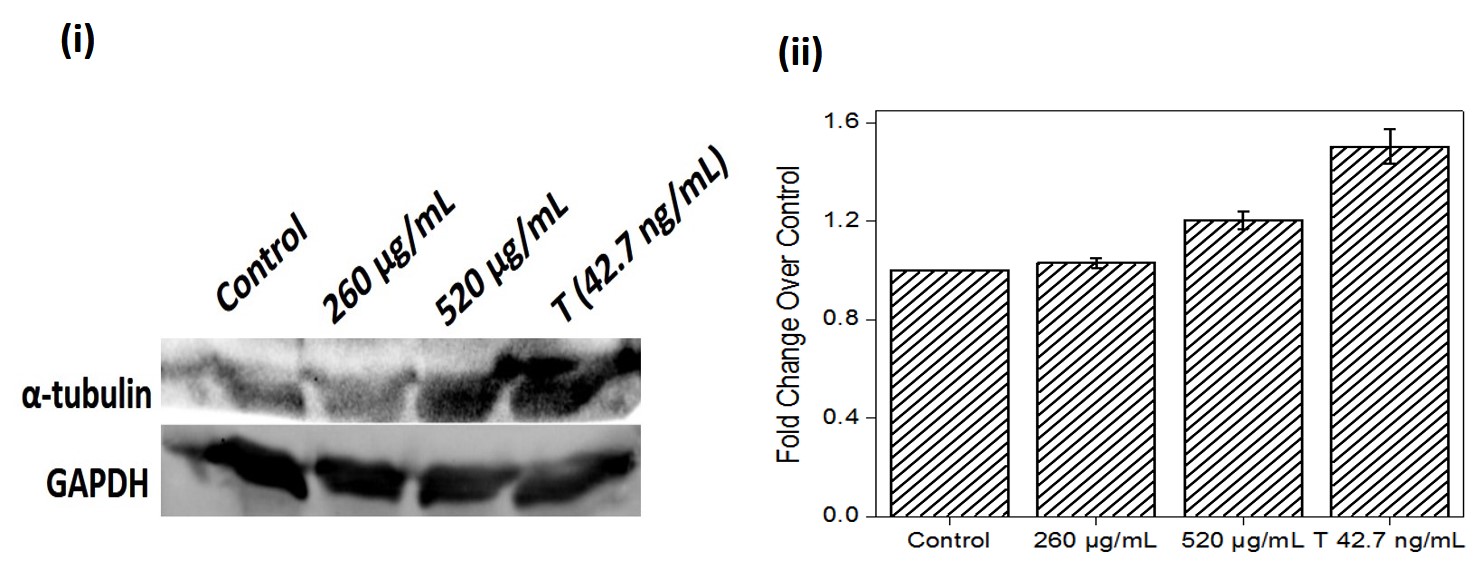
**

**Figure S4**


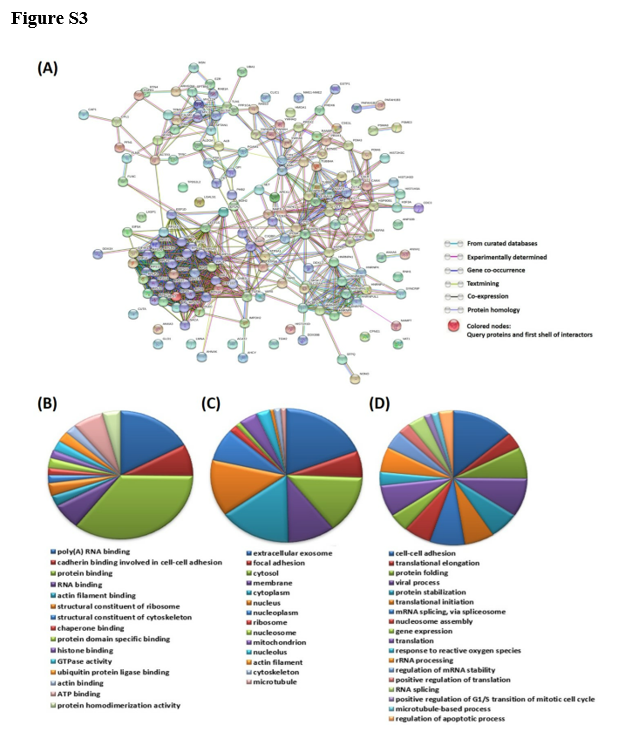


**Figure S5**


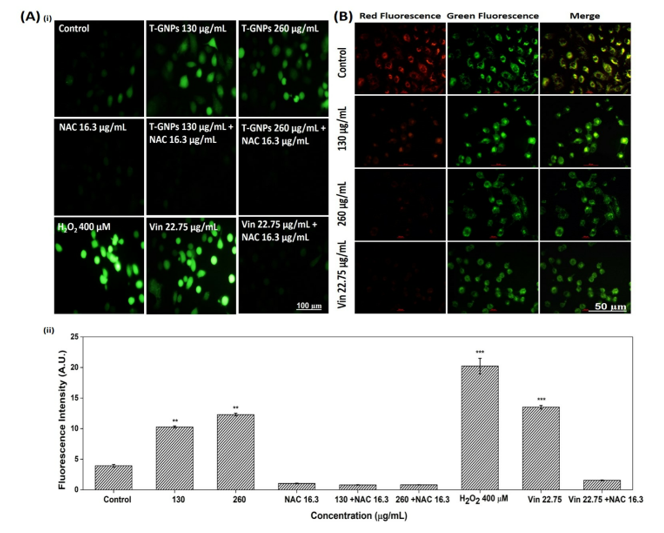


**Figure S6**


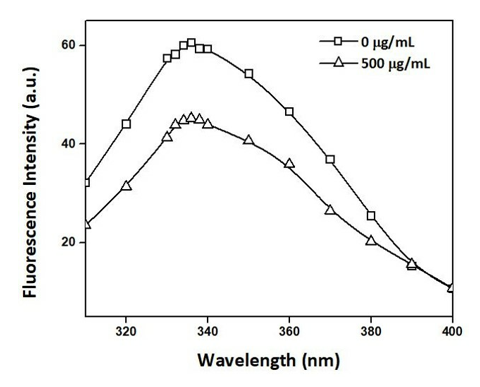


**Figure S7.** Full-length blot images at different exposures shown in Figure 3B (iii)


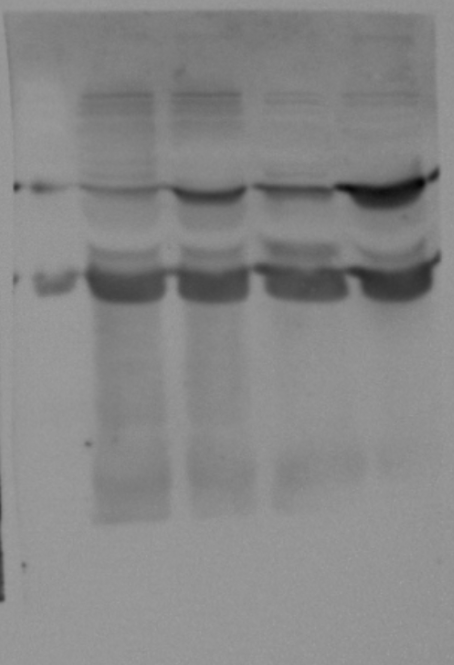

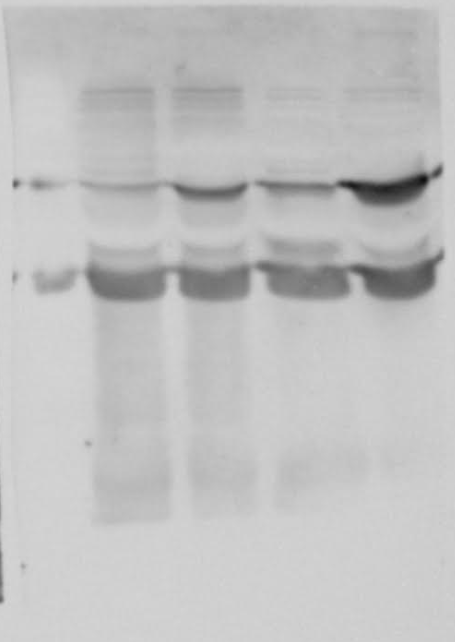


Full-length blots were cut into two to stain for alpha tubulin and GAPDH antibodies as shown in Figure S3


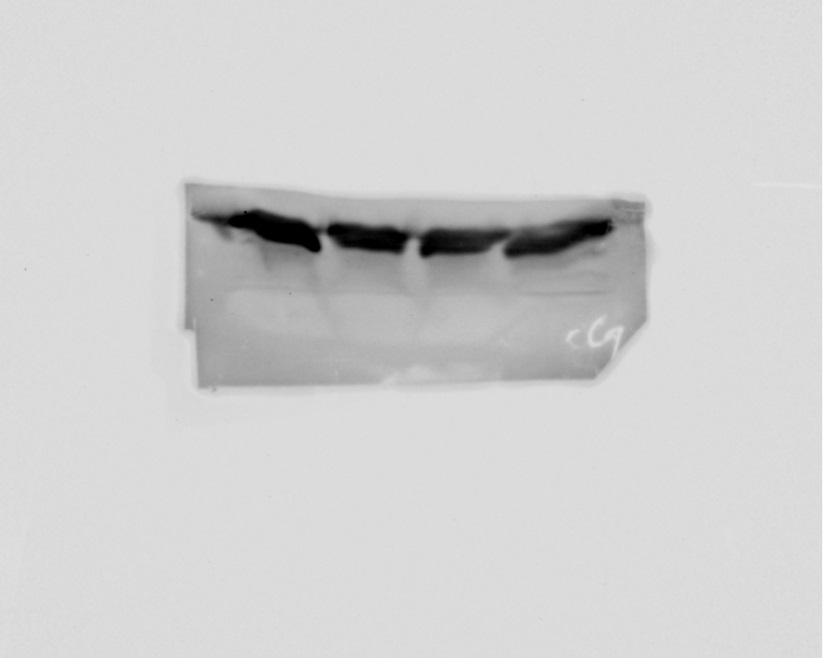

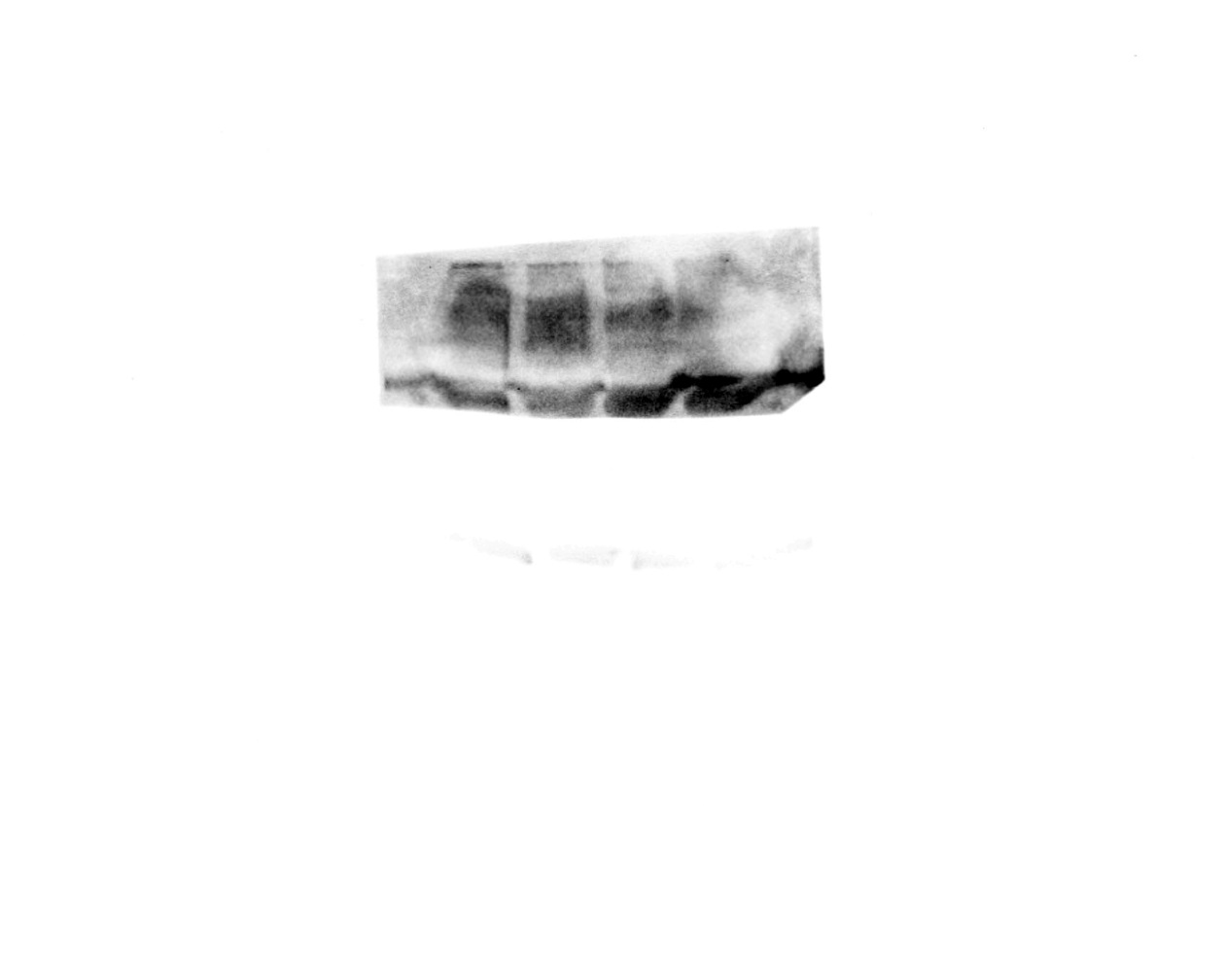


**Table S1**

| **Uniprot ID** | **Protein Name** | **Peptide spectrum matches** | **Protein sequence coverage (%)** | **Summed morpheus Score** | **MW (Dalton)** | **PI** |
| --- | --- | --- | --- | --- | --- | --- |
| A0A087WUZ3 | Spectrin beta chain | 2 | 1.141 | 19.065 | 274,829 | 5.39 |
| A0A087WYR3 | Tumor protein D54 | 4 | 19.81 | 31.1036831 | 23,765 | 6.13 |
| A0A0A0MTS2 | Glucose-6-phosphate isomerase | 4 | 5.58 | 35.1147563 | 64,825 | 9.10 |
| A0A0D9SF53 | ATP-dependent RNA helicase DDX3X | 2 | 4.91 | 27.062226 | 81,477 | 8.29 |
| A0A0D9SGF6 | Spectrin alpha chain, non-erythrocytic 1 | 2 | 1.56 | 36.0621264 | 287,605 | 5.24 |
| A0A0U1RRH7 | Histone H2A | 14 | 20.58 | 43.5827097 | 18,553 | 11.52 |
| B0QZ18 | Copine-1 | 6 | 12.17 | 57.1709055 | 59,718 | 5.66 |
| B7Z6Z4 | Myosin light polypeptide 6 | 4 | 15.54 | 30.2095708 | 26,707 | 5.01 |
| E7EMB3 | Calmodulin-2 | 8 | 28.06 | 47.2399197 | 21,689 | 4.45 |
| E9PAV3 | Nascent polypeptide-associated complex subunit alpha, muscle-specific form | 5 | 2.02 | 37.1448829 | 205,422 | 9.60 |
| E9PK25 | Cofilin-1 | 27 | 34.31 | 81.4162836 | 22,728 | 8.51 |
|  |  |  |  |  |  |  |
| E9PRY8 | Elongation factor 1-delta | 9 | 5.30 | 41.1530769 | 76,570 | 6.61 |
| F6WQW2 | Ran-specific GTPase-activating protein | 2 | 12.23 | 18.0961841 | 31,904 | 8.96 |
| G8JLB6 | Heterogeneous nuclear ribonucleoprotein H | 8 | 9.95 | 48.2235464 | 51,230 | 6.33 |
| I3L504 | Eukaryotic translation initiation factor 5A-1 | 15 | 19.35 | 44.2456568 | 20,504 | 5.09 |
| J3KN67 | Tropomyosin alpha-3 chain | 11 | 19.64 | 71.4483463 | 33,222 | 4.73 |
| J3KQE5 | GTP-binding nuclear protein Ran | 4 | 16.23 | 35.200498 | 26,816 | 9.62 |
| J3QQX2 | Rho GDP-dissociation inhibitor 1 | 4 | 13.19 | 30.217245 | 25,831 | 6.93 |
| J3QRS3 | Myosin regulatory light chain 12A | 3 | 16.38 | 30.1011801 | 20,457 | 4.60 |
| M0R0R2 | 40S ribosomal protein S5 | 11 | 12.44 | 41.1781301 | 25,333 | 9.78 |
| O00299 | Chloride intracellular channel protein 1 | 12 | 38.58 | 77.3850311 | 26,923 | 5.09 |
| O60506 | Heterogeneous nuclear ribonucleoprotein Q | 4 | 4.17 | 28.0596779 | 69,603 | 8.68 |
| O60888 | Protein CutA | 5 | 22.90 | 28.0615921 | 19,116 | 5.42 |
| P00441 | Superoxide dismutase [Cu-Zn] | 5 | 38.96 | 44.1474032 | 15,936 | 5.70 |
| P00558 | Phosphoglycerate kinase 1 | 22 | 31.41 | 103.4812 | 44,615 | 8.30 |
| P02545 | Prelamin-A/C | 6 | 6.32 | 38.2500606 | 74,139 | 6.57 |
| P02768 | Serum albumin | 10 | 5.91 | 37.649066 | 69,367 | 5.92 |
| P02786 | Transferrin receptor protein 1 | 5 | 3.81 | 27.0732444 | 84,871 | 6.18 |
| P04075 | Fructose-bisphosphate aldolase A | 6 | 17.03 | 39.2115364 | 39,420 | 8.30 |
| P04083 | Annexin A1 | 19 | 27.16 | 96.4296179 | 38,714 | 6.57 |
| P04350 | Tubulin beta-4A chain | 91 | 56.98 | 232.290711 | 49,586 | 4.78 |
| P04792 | Heat shock protein beta-1 | 2 | 13.17 | 19.1189725 | 22,783 | 5.98 |
| P05386 | 60S acidic ribosomal protein P1 | 8 | 51.75 | 40.1879504 | 11,514 | 4.21 |
| P05387 | 60S acidic ribosomal protein P2 | 16 | 69.56 | 63.4221054 | 11,665 | 4.38 |
| P05388 | 60S acidic ribosomal protein P0 | 6 | 16.71 | 45.145162 | 34,273 | 5.70 |
| P06576 | ATP synthase subunit beta, mitochondrial | 12 | 15.12 | 74.2943906 | 56,560 | 5.26 |
| P06748 | Nucleophosmin | 22 | 24.48 | 60.4587964 | 32,575 | 4.64 |
| P07737 | Profilin-1 | 17 | 40 | 58.3860217 | 15,054 | 8.44 |
| P08133 | Annexin A6 | 17 | 16.04 | 111.601955 | 75,873 | 5.41 |
| P09211 | Glutathione S-transferase P | 11 | 26.19 | 52.1721036 | 23,356 | 5.43 |
| P09382 | Galectin-1 | 7 | 26.66 | 41.328999 | 14,716 | 5.3 |
| P09651 | Heterogeneous nuclear ribonucleoprotein A1 | 8 | 6.98 | 28.1420167 | 38,747 | 9.17 |
| P0C0S5 | Histone H2A.Z | 4 | 35.15 | 27.4617474 | 13,553 | 10.58 |
| P10599 | Thioredoxin | 6 | 22.85 | 23.1649953 | 11,737 | 4.82 |
| P10809 | 60 kDa heat shock protein, mitochondrial | 20 | 20.76 | 103.494269 | 61,055 | 5.70 |
| P12004 | Proliferating cell nuclear antigen | 4 | 15.32 | 29.1006853 | 28,769 | 4.57 |
| P12235 | ADP/ATP translocase 1 | 3 | 13.08 | 24.1237222 | 33,064 | 9.78 |
| P12268 | Inosine-5'-monophosphate dehydrogenase 2 | 2 | 6.80 | 22.0469375 | 55,805 | 6.44 |
| P12429 | Annexin A3 | 3 | 7.73 | 21.0831378 | 36,375 | 5.62 |
| P12814 | Alpha-actinin-1 | 38 | 17.60 | 175.014706 | 103,057 | 5.25 |
| P13489 | Ribonuclease inhibitor | 2 | 7.37 | 22.0698711 | 49,973 | 4.71 |
| P14625 | Endoplasmin | 4 | 4.48 | 27.1331004 | 92,469 | 4.76 |
| P15311 | Ezrin | 7 | 6.14 | 36.3678183 | 69,413 | 5.94 |
| P16104 | Histone H2AX | 9 | 27.27 | 36.4703116 | 15,145 | 10.74 |
| P16402 | Histone H1.3 | 6 | 14.9 | 38.3873621 | 22,350 | 11.02 |
| P17066 | Heat shock 70 kDa protein 6 | 8 | 6.22 | 38.5376595 | 71,028 | 5.81 |
| P17096 | High mobility group protein HMG-I/HMG-Y | 2 | 23.36 | 20.0654008 | 11,676 | 10.31 |
| P18669 | Phosphoglycerate mutase 1 | 14 | 30.31 | 63.5389369 | 28,804 | 6.67 |
| P19338 | Nucleolin | 4 | 7.46 | 39.1142316 | 76,614 | 4.60 |
| P21333 | Filamin-A | 27 | 9.59 | 173.945197 | 280,737 | 5.70 |
| P21980 | Protein-glutamine gamma-glutamyltransferase 2 | 23 | 18.63 | 109.67288 | 77,329 | 5.11 |
| P22314 | Ubiquitin-like modifier-activating enzyme 1 | 11 | 6.99 | 74.231028 | 117,849 | 5.49 |
| P22626 | Heterogeneous nuclear ribonucleoproteins A2/B1 | 5 | 15.86 | 44.2080501 | 37,430 | 8.97 |
| P23246 | Splicing factor, proline- and glutamine-rich | 5 | 5.51 | 28.0794811 | 76,149 | 9.45 |
| P23526 | Adenosylhomocysteinase | 13 | 12.03 | 48.2866713 | 47,716 | 5.92 |
| P24534 | Elongation factor 1-beta | 11 | 20.88 | 39.178672 | 24,764 | 4.50 |
| P25705 | ATP synthase subunit alpha, mitochondrial | 7 | 10.48 | 51.2752111 | 59,751 | 9.16 |
| P26038 | Moesin | 36 | 23.91 | 170.012268 | 67,820 | 6.08 |
| P26639 | Threonine--tRNA ligase, cytoplasmic | 3 | 6.22 | 32.1237798 | 83435 | 6.23 |
| P26640 | Valine--tRNA ligase | 2 | 2.05 | 20.0754476 | 272288 | 7.50 |
| P26641 | Elongation factor 1-gamma | 13 | 13.04 | 52.3234631 | 50118 | 6.25 |
| P27348 | 14-3-3 protein theta | 21 | 20 | 62.5878641 | 27764 | 4.68 |
| P27695 | DNA-(apurinic or apyrimidinic site) lyase | 5 | 11.00 | 22.0857471 | 35554 | 8.33 |
| P27797 | Calreticulin | 8 | 13.18 | 37.1553643 | 48141 | 4.29 |
| P27824 | Calnexin | 5 | 5.23 | 27.0689645 | 67568 | 4.46 |
| P29401 | Transketolase | 18 | 12.03 | 66.6022042 | 117769 | 7.93 |
| P30041 | Peroxiredoxin-6 | 5 | 18.75 | 33.2119697 | 25034 | 6.00 |
| P30101 | Protein disulfide-isomerase A3 | 4 | 6.93 | 26.1034706 | 56782 | 5.98 |
| P31946 | 14-3-3 protein beta/alpha | 17 | 2.35 | 61.5341843 | 28082 | 4.76 |
| P31948 | Stress-induced-phosphoprotein 1 | 7 | 7.73 | 37.1518691 | 62639 | 6.40 |
| P32119 | Peroxiredoxin-2 | 7 | 14.64 | 37.1571373 | 21891 | 5.66 |
| P35268 | 60S ribosomal protein L22 | 3 | 30.46 | 29.1382005 | 14786 | 9.21 |
| P35579 | Myosin-9 | 60 | 16.63 | 321.288275 | 226532 | 5.50 |
| P36578 | 60S ribosomal protein L4 | 4 | 7.02 | 25.2155653 | 47697 | 11.07 |
| P37108 | Signal recognition particle 14 kDa protein | 4 | 31.61 | 32.1760234 | 14569 | 10.05 |
| P38646 | Stress-70 protein, mitochondrial | 17 | 15.90 | 90.4456328 | 73680 | 5.87 |
| P40926 | Malate dehydrogenase, mitochondrial | 21 | 28.40 | 85.3626052 | 35503 | 8.92 |
| P43490 | Nicotinamide phosphoribosyltransferase | 6 | 12.01 | 38.1122184 | 55521 | 6.69 |
| P48643 | T-complex protein 1 subunit epsilon | 6 | 6.09 | 29.0658947 | 225958 | 5.50 |
| P49368 | T-complex protein 1 subunit gamma | 10 | 13.57 | 58.2612641 | 116190 | 5.75 |
| P49411 | Elongation factor Tu, mitochondrial | 5 | 11.28 | 47.1751004 | 49541 | 7.26 |
| P50395 | Rab GDP dissociation inhibitor beta | 7 | 14.38 | 49.2106165 | 50663 | 6.10 |
| P50990 | T-complex protein 1 subunit theta | 3 | 5.83 | 31.153192 | 59620 | 5.41 |
| P50991 | T-complex protein 1 subunit delta | 3 | 4.82 | 20.1005101 | 57924 | 7.96 |
| P52597 | Heterogeneous nuclear ribonucleoprotein F | 5 | 8.19 | 30.1168666 | 45671 | 5.37 |
| P55060 | Exportin-2 | 3 | 3.91 | 26.118891 | 110416 | 5.51 |
| P55072 | Transitional endoplasmic reticulum ATPase | 17 | 19.60 | 119.548452 | 89321 | 5.14 |
| P55209 | Nucleosome assembly protein 1-like 1 | 9 | 17.13 | 42.2208993 | 228368 | 4.45 |
| P60842 | Eukaryotic initiation factor 4A-I | 5 | 8.37 | 34.2384118 | 46153 | 5.32 |
| P61026 | Ras-related protein Rab-10 | 3 | 11 | 20.103208 | 22540 | 8.58 |
| P61158 | Actin-related protein 3 | 3 | 9.80 | 28.1153752 | 89357 | 5.51 |
| P61289 | Proteasome activator complex subunit 3 | 3 | 11.81 | 31.0750514 | 96788 | 5.52 |
| P61604 | 10 kDa heat shock protein, mitochondrial | 5 | 21.56 | 20.2481423 | 16103 | 5.53 |
| P61978 | Heterogeneous nuclear ribonucleoprotein K | 16 | 17.71 | 75.4728212 | 50976 | 5.39 |
| P62081 | 40S ribosomal protein S7 | 6 | 17.52 | 26.1517152 | 43420 | 10.21 |
| P62136 | Serine/threonine-protein phosphatase PP1-alpha catalytic subunit | 3 | 14.54 | 33.1219426 | 66391 | 5.18 |
| P62241 | 40S ribosomal protein S8 | 2 | 13.46 | 21.089167 | 46066 | 10.37 |
| P62258 | 14-3-3 protein epsilon | 26 | 27.84 | 88.8143472 | 29173 | 4.63 |
| P62318 | Small nuclear ribonucleoprotein Sm D3 | 3 | 15.07 | 21.1940605 | 13916 | 10.33 |
| P62424 | 60S ribosomal protein L7a | 3 | 10.90 | 35.095666 | 51522 | 10.80 |
| P62701 | 40S ribosomal protein S4, X isoform | 3 | 10.26 | 21.1064714 | 29597 | 10.16 |
| P62820 | Ras-related protein Rab-1A | 4 | 13.17 | 24.1135781 | 66829 | 5.52 |
| P63244 | Receptor of activated protein C kinase 1 | 4 | 13.56 | 34.1424826 | 117212 | 7.44 |
| P67936 | Tropomyosin alpha-4 chain | 1 | 22.58 | 62.3566145 | 28521 | 4.67 |
| P68363 | Tubulin alpha-1B chain | 78 | 32.15 | 161.536006 | 50151 | 4.94 |
| P68402 | Platelet-activating factor acetylhydrolase IB subunit beta | 5 | 12.2 | 21.2345286 | 25569 | 5.57 |
| P68431 | Histone H3.1 | 3 | 30.14 | 22.2012943 | 15404 | 11.13 |
| P78371 | T-complex protein 1 subunit beta | 11 | 12.71 | 44.185776 | 159071 | 6.04 |
| P84243 | Histone H3.3 | 3 | 30.14 | 19.1982574 | 30224 | 11.35 |
| Q00839 | Heterogeneous nuclear ribonucleoprotein U | 6 | 4 | 27.1368981 | 90584 | 5.76 |
| Q01105 | Protein SET | 8 | 23.10 | 49.3300063 | 64594 | 4.17 |
| Q01518 | Adenylyl cyclase-associated protein 1 | 5 | 7.57 | 35.1492854 | 202648 | 7.23 |
| Q04760 | Lactoylglutathione lyase | 5 | 25.54 | 35.1278542 | 20777 | 5.12 |
| Q04917 | 14-3-3 protein eta | 9 | 13.41 | 35.4280063 | 28218 | 4.76 |
| Q05639 | Elongation factor 1-alpha 2 | 15 | 12.74 | 47.6042629 | 50470 | 9.11 |
| Q06830 | Peroxiredoxin-1 | 7 | 15.57 | 32.2575786 | 41068 | 7.59 |
| Q07021 | Complement component 1 Q subcomponent-binding protein, mitochondrial | 14 | 21.63 | 65.3459337 | 31362 | 4.74 |
| Q09666 | Neuroblast differentiation-associated protein AHNAK | 2 | 3.66 | 42.1620158 | 629101 | 5.80 |
| Q13838 | Spliceosome RNA helicase DDX39B | 4 | 4.67 | 19.2665699 | 146910 | 5.76 |
| Q14103 | Heterogeneous nuclear ribonucleoprotein D0 | 4 | 6.19 | 23.2214304 | 127470 | 8.84 |
| Q14315 | Filamin-C | 13 | 3.41 | 77.3643221 | 291022 | 5.65 |
| Q14847 | LIM and SH3 domain protein 1 | 3 | 10.72 | 21.091688 | 29717 | 6.61 |
| Q14974 | Importin subunit beta-1 | 15 | 11.98 | 87.3357486 | 97170 | 4.68 |
| Q15084 | Protein disulfide-isomerase A6 | 8 | 8.86 | 33.1150344 | 48121 | 4.95 |
| Q15102 | Platelet-activating factor acetylhydrolase IB subunit gamma | 2 | 11.5 | 19.2046865 | 25734 | 6.33 |
| Q15149 | Plectin | 5 | 1.23 | 43.2049194 | 531790 | 5.74 |
| Q15233 | Non-POU domain-containing octamer-binding protein | 5 | 8.28 | 26.0882807 | 77886 | 9.43 |
| Q15366 | Poly(rC)-binding protein 2 | 5 | 7.94 | 26.1149551 | 120923 | 8.27 |
| Q16543 | Hsp90 co-chaperone Cdc37 | 6 | 10.05 | 26.0783986 | 44468 | 5.17 |
| Q16629 | Serine/arginine-rich splicing factor 7 | 3 | 8.82 | 20.0828058 | 70021 | 11.64 |
| Q32Q12 | Nucleoside diphosphate kinase | 19 | 29.10 | 59.3201587 | 49921 | 8.76 |
| Q5HYB6 | Epididymis luminal protein 189 | 11 | 24.56 | 68.4317034 | 53578 | 4.73 |
| Q6IPX4 | 40S ribosomal protein S16 | 3 | 13.81 | 19.1472672 | 73369 | 10.39 |
| Q71DI3 | Histone H3.2 | 4 | 30.14 | 25.2054457 | 15388 | 11.27 |
| Q8TAA3 | Proteasome subunit alpha-type 8 | 2 | 14.06 | 21.0838828 | 56398 | 8.95 |
| Q92688 | Acidic leucine-rich nuclear phosphoprotein 32 family member B | 5 | 10.75 | 32.1591086 | 28787 | 3.93 |
| Q92841 | Probable ATP-dependent RNA helicase DDX17 | 2 | 3.42 | 23.1154024 | 218471 | 8.93 |
| Q99536 | Synaptic vesicle membrane protein VAT-1 homolog | 2 | 7.63 | 24.085079 | 41920 | 5.88 |
| Q99623 | Prohibitin-2 | 5 | 13.71 | 33.125603 | 96223 | 9.89 |
| Q9BUF5 | Tubulin beta-6 chain | 45 | 26.00 | 99.5538378 | 49857 | 4.77 |
| Q9BUJ2 | Heterogeneous nuclear ribonucleoprotein U-like protein 1 | 2 | 4.2 | 23.0654441 | 339358 | 8.80 |
| Q9BWD1 | Acetyl-CoA acetyltransferase, cytosolic | 5 | 15.36 | 41.2504982 | 41350 | 6.46 |
| Q9NQC3 | Reticulon-4 | 3 | 5.62 | 34.1308635 | 129931 | 4.42 |
| Q9Y490 | Talin-1 | 15 | 4.95 | 118.307673 | 269767 | 5.77 |
| S4R435 | RPS10-NUDT3 readthrough | 8 | 8.39 | 24.1931282 | 71326 | 9.78 |
